# Overexpression and potential roles of NRIP1 in psoriasis

**DOI:** 10.18632/oncotarget.12371

**Published:** 2016-09-30

**Authors:** Chao Luan, Xu Chen, Yu Hu, Zhimin Hao, Jared M. Osland, Xundi Chen, Skyler D. Gerber, Min Chen, Heng Gu, Rong Yuan

**Affiliations:** ^1^ Jiangsu Key Laboratory of Molecular Biology for Skin Diseases and STIs, Institute of Dermatology, Chinese Academy of Medical Sciences & Peking Union Medical College, Nanjing, Jiangsu, China; ^2^ Department of Internal Medicine, Southern Illinois University School of Medicine, Springfield, IL, USA; ^3^ Department of Medical Microbiology, Immunology and Cell Biology, Southern Illinois University School of Medicine, Springfield, IL, USA

**Keywords:** NRIP1, psoriasis, skin, T cells, animal model

## Abstract

Nuclear receptor interacting protein 1 (NRIP1, also known as RIP140) is a co-regulator for various transcriptional factors and nuclear receptors, and has been shown to take part in many biological and pathological processes, such as regulating mammary gland development and inflammatory response.

The aim of this study is to investigate the expression of NRIP1 and to explore its roles in the pathogenesis of psoriasis. Thirty active psoriasis patients and 16 healthy volunteers were enrolled for this study. qRT-PCR analyses found that both NRIP1 and RelA/p65 were elevated in psoriatic lesions compared to psoriatic non-lesions and normal controls, and also overexpressed in peripheral blood mononuclear cell (PBMCs) of psoriasis patients. Suppression of NRIP1 in HaCaT cells could significantly inhibit cell growth and induce apoptosis, and the suppression of NRIP1 in CD4^+^ T cells isolated from psoriasis patients could downregulate the expression of RelA/p65 and decrease the secretion of IL-17. Furthermore, in *Nrip1* knockout mice, IMQ-induced inflammation of skin was delayed and the RelA/p65 expression in lesions was reduced. In conclusion, our data suggests that NRIP1 is overexpressed both in skin and PBMCs of psoriasis patients and may be involved in the abnormal proliferation and apoptosis of keratinocytes, as well as the immune reaction through the regulation of RelA/p65. Therefore, NRIP1 may be a potential therapeutic target for psoriasis.

## INTRODUCTION

NRIP1, one of the first isolated nuclear receptor transcriptional cofactors, works as an unconventional co-regulator for many nuclear receptors. It is highly expressed in adipose tissue [1], liver [2] and skeletal muscle and mainly acts as a transcriptional co-repressor of genes involved in glucose uptake, glycolysis, fatty acid oxidation, mitochondrial biogenesis and oxidative phosphorylation to ultimately repress energy utilization [3]. However, accumulating studies have provided evidence that NRIP1 may also work as a co-activator for a variety of other transcription factors in numerous cellular responses, such as inflammatory gene expression in macrophages [4], amphiregulin expression in the ovary [5] and triglyceride synthesis in the liver [2]. In macrophages, NRIP1 functions as a co-activator for NF-κB through direct interaction with RelA/p65 and the transcriptional co-activator CREB-binding protein (CBP), and up-regulates the downstream inflammatory gene expression such as TNF-α and interleukin-6 [4]. In addition, a study also showed that NRIP1 expression was controlled by the E2 promotor binding factor (E2F) pathway, which may play a crucial role in gene transcription, cellular proliferation and apoptosis [6]. We recently reported that NRIP1 was overexpressed in human breast cancer and the suppression of NRIP1 could inhibit growth and induce apoptosis of breast cancer cells [7].

Psoriasis is a chronic inflammatory skin disorder affecting approximately 1-3% of the general population. It is characterized by keratinocytes hyper-proliferation and inflammatory cellular infiltrate in both dermis and epidermis. NF-κB is identified as a key mediator in the pathogenesis of psoriasis because of its involvement in inflammatory pathways, proliferation and apoptosis of various cell types [8]. Previous studies have elucidated that NF-κB levels are elevated in both psoriatic plaques (PP), and uninvolved, clinically asymptomatic skin of psoriatic patients (PN) compared to normal healthy skin (NN). Numerous chemokines and cytokines in the pathogenesis of psoriasis are dependent on the NF-κB signaling pathway [9]. NF-κB functions as a link between abnormal keratinocytes and immune cell states [10]. Various treatments that improve the symptoms of psoriasis, including epigallocatechin-3-gallate (EGCG), IL-12/23 and TNF-α inhibitors, could also suppress the expression or activity of NF-κB [9].

In order to evaluate the function of NRIP1 in cell growth, apoptosis and inflammation involved in psoriasis, we used human skin biopsies and peripheral blood mononuclear cells (PBMCs), HaCaT cells and CD4^+^ T cells isolated from psoriasis patients, along with an *in vivo* study of IMQ-induced psoriasis in *Nrip1* knockout mice. Our results suggest that NRIP1 may play a role in the pathogenesis of psoriasis and may be a novel therapeutic target for psoriasis.

## RESULTS

### Overexpression of NRIP1and p65 in lesions and PBMCs of psoriasis patients

**Figure 1:**
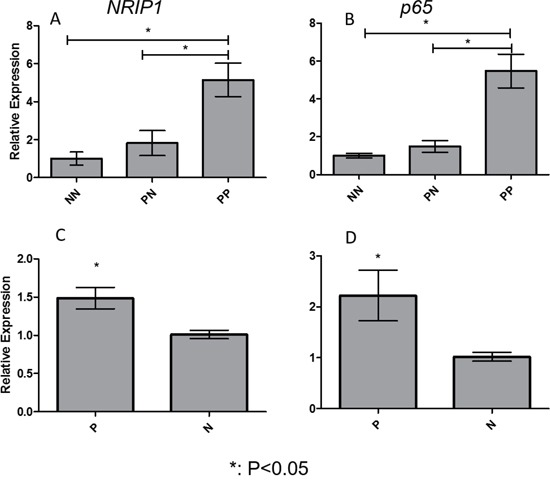
Expression of NRIP1 and p65 was elevated in lesions and PBMCs of psoriasis patients Expression of *NRIP1* mRNA **A.** and *p65* mRNA **B.** was significantly increased in PP compared to PN and NN, respectively (p<0.05). In PBMCs, the expression of *NRIP1* mRNA **C.** and *p65* mRNA **D.** was remarkably elevated in psoriasis patients (p<0.05). PP: psoriatic lesions, PN: psoriatic non-lesions, NN: healthy controls, P: psoriasis patients, N: Normal persons

In order to investigate its potential role in psoriasis, we first measured the expression of NRIP1 in the skin lesions and PBMCs of psoriasis patients. QRT-PCR assays showed that mRNA of *NRIP1* significantly increased in PP compared to PN (5.1430 ± 0.8793 vs. 1.8170 ± 0.6592, P=0.0218), but no significant difference was observed between PN and NN (1.817 ± 0.6592 vs. 1.000 ± 0.3413, P=0.4880) (Figure 1A). The same trend was found in the p65 expression, which is significantly increased in PP compared to PN (5.4670 ± 0.8819 vs. 1.4870 ± 0.3091, P= 0.0006); but no significant difference was found between in PN and in NN (1.4870 ± 0.3091 vs. 1.000 ± 0.1241, P=0.4023) (Figure 1B). There was no significant correlation between the mRNA expression of NRIP1 and p65 in psoriatic lesions (r = −0.013, P=0.974).

The previous study by our team also applied immunohistochemistry (IHC) on three types of skin biopsies, and found that expression of NRIP1 was significantly increased in both the epidermis and dermis of PP. NRIP1 positive cells were widely distributed in epidermis (keratinocytes and melanocytes) and dermis (lymphocytes, fibroblasts and epithelia cells), but not in the stratum corneum. There was no significant difference between PN and NN groups either in epidermis or dermis(data unpublished).

Expression of NRIP1 and p65 were also significantly elevated in PBMCs of psoriasis patients compared to healthy controls (1.4860 ± 0.1408 vs. 1.0110 ± 0.05578, P= 0.0165 & 2.2220 ± 0.4975 vs. 1.019 ± 0.08736, P= 0.0385, respectively. Figure 1C & 1D).

### Suppression of NRIP1 inhibited growth and induced apoptosis of HaCaT cells

**Figure 2:**
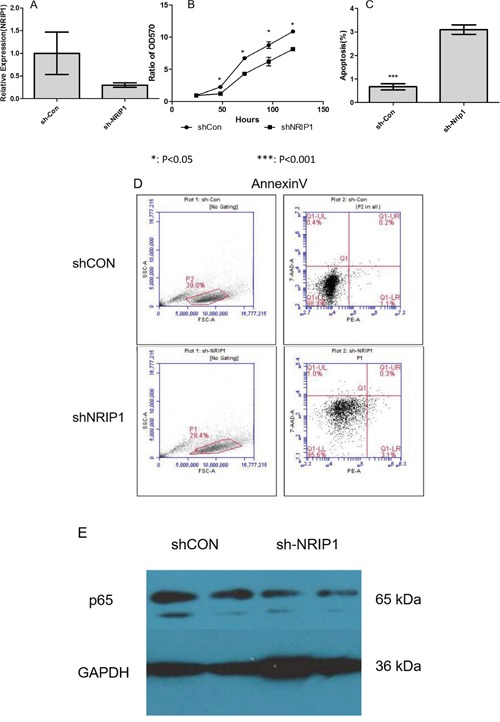
Suppression of NRIP1 inhibited the growth and induced apoptosis of HaCaT cells **A.** shNRIP1 suppressed the NRIP1 expression in HaCaT cells. **B.** MTT assay showed shNRIP1 reduced the growth of HaCaT cells significantly at the 48h, 72h, 96h and 120h time points (p<0.05) after being seeded, respectively. **C&D**. Annexin V showed that shNRIP1 induced the apoptosis of HaCaT cells significantly (p<0.001). **E.** Western blot result showed that the expression of p65 was remarkably reduced by shNRIP1 transfection in HaCaT cells.

Excessive proliferation of keratinocytes is an important pathological feature of psoriasis, and most of the treatments that improve the condition of psoriasis could suppress the proliferation of the keratinocytes. In order to illustrate the *in vitro* significance of NRIP1 on the proliferation of keratinocytes, we applied shRNA targeting *NRIP1* (shNRIP1) to suppress *NRIP1* expression in HaCaT cells, and used non sense shRNA (shCON). The qRT-PCR results (Figure 2A) showed a 70% depletion of *NRIP1* expression in HaCaT cells (mRNA expression of shNRIP1 1.0±0.66, shCON 0.30±0.07). These HaCat cells, transfected with shNRIP1 and shCON, were used for the next experiments.

As the 3-(4,5-dimethylthiazol-2-yl)-2,5-diphnyltetrazolium bromide (MTT) assay showed (Figure 2B), shNRIP1 reduced the growth of HaCaT cells significantly at the 48h, 72h, 96h and 120h time points after being seeded (P<0.05).

In order to illustrate the reasons for the reduction of cell growth by suppressing NRIP1, we detected cell proliferation and apoptosis of HaCaT cells by PI staining assay and Annexin-V assay and flow cytometry. As shown in Figure 2C&2D, suppressing NRIP1 remarkably induced apoptosis of HaCat cells (P<0.05); however, there is no clear trend of alteration in G0/G1, S and G2/M phases between shCON and shNRIP1 treatment of HaCaT cells. These results suggest that suppression of NRIP1 could induce apoptosis of HaCaT cells.

As the above data showed that both NRIP1 and p65 were overexpressed in psoriasis lesions, we applied western blot assay to detect the function of silencing NRIP1 on the expression of p65 in HaCaT cells. The western blot data (Figure 2E) showed that the expression of p65 was reduced significantly by the suppression of NRIP1 in HaCaT cells.

### Suppression of NRIP1 reduced expression of p65 and decreased secretion of IL-17 in CD4+T cells

**Figure 3:**
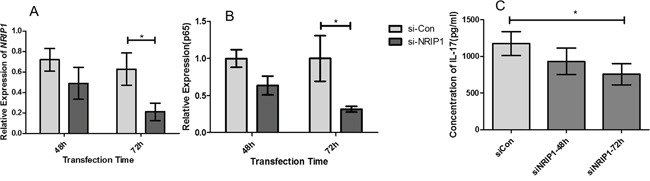
Suppression of NRIP1 reduced expression of p65 and decreased secretion of IL-17 in CD4+T cells QRT-PCR assay showed that the expression of *NRIP1* mRNA **A.** and *p65* mRNA **B.** in CD4^+^T cells were reduced significantly at 72h after siNRIP1 treatment (P<0.05). ELISA assay **C.** showed siNRIP1 transfection decreased the concentration of IL-17 in the supernatants of CD4^+^T cells at 72h after siNRIP1 treatment (p<0.05).

CD4+ T cells are the major resource of the inflammatory cytokines, such as IL-17. To investigate the function of NRIP1 in regulating the secretary profile of CD4+ T cells, we isolated CD4+ T cells from psoriasis patients and used siRNA to silence NRIP1 (siNRIP1) expression. As examined by qRT-PCR at 48h and 72h after siRNA treatment (Figure 3A), the most significant *NRIP1* mRNA inhibition of siNRIP1 treatment compared to siCON treatment (P<0.05) was detected at 72h. We then measured the expression of *p65* mRNA at the same time points and found the suppression of NRIP1 downregulated the expression of *p65* mRNA at each time point. The most significant reduction occurred at 72h, which is in accordance with the expression of *NRIP1* mRNA (Figure 3B). The supernatants of culture medium were also collected for ELISA. The result showed that the concentration of IL-17 was significantly decreased at 72h (p<0.05, Figure 3C).

### The depletion of *Nrip1* delays IMQ-induced skin inflammation *in vivo*

**Figure 4:**
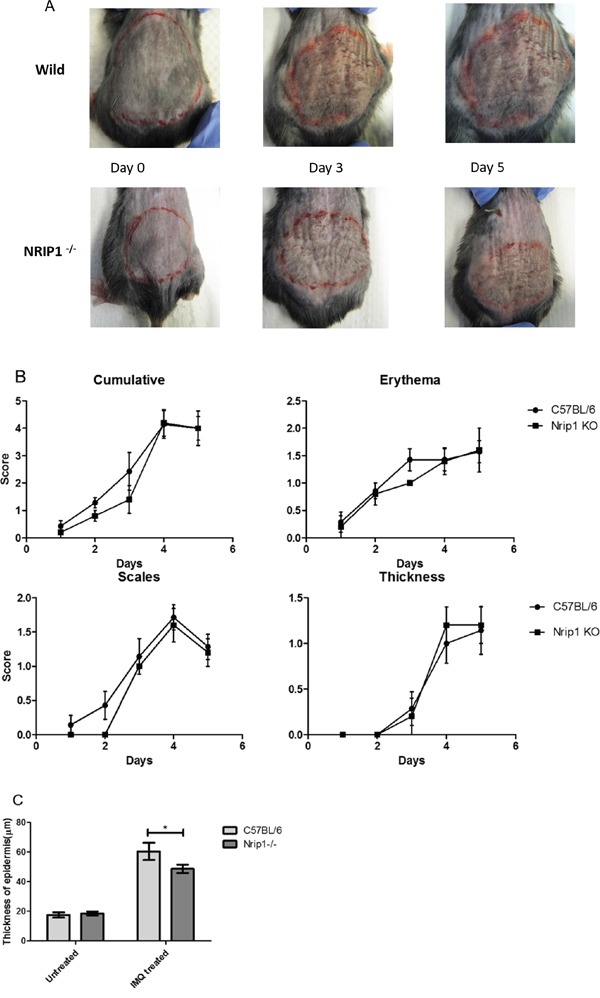
Nrip1 deficiency could delay the IMQ-induced skin inflammation **A.** at the third day of IMQ treatment, the skin inflammation of Nrip1^−/−^ mice was weaker than C57BL/6 WT mice, but at the fifth day, C57BL/6 WT and *Nrip1^−/−^* mice developed similar skin inflammation resembling psoriasis. **B.** compared with C57BL/6 WT mice, the scores of erythema and scaling of *Nrip1^−/−^* mice were lower before the third day. From the fourth day, scores of erythema and scaling of both groups are similar. Cumulative scores showed the same trend. There was no difference of thickness scores between the two groups. **C.** measurement of the thickness of epidermis in H&E stained skin sections showed that after 5 days of IMQ treatment, thickness of epidermis was increased in both two groups, but the increment was significantly reduced in *Nrip1^−/−^*mice.

Overexpression of NRIP1 in the tissues of patients and the effects of silencing *NRIP1* in HaCaT cells strongly suggested that the depletion of NRIP1 may improve the skin lesion of psoriasis. To test this hypothesis, we compared the psoriasis-like reactions induced by IMQ treatment between wild-type (WT) B6 and *Nrip1−/−* mice. Mice were treated with 5% IMQ cream on their shaved backs for five consecutive days. Two or three days after application, signs of erythema, scaling, and thickness began to occur. On the third day, indicated by erythema and scaling, the skin inflammation of *Nrip1−/−* mice was weaker than WT mice. By the fifth day, both groups of mice developed similar lesions resembling psoriasis (Figure 4A). The independent scores and cumulative scores are depicted in Figure 4B. Compared with WT mice, the scores of erythema and scaling of *Nrip1−/−* mice were lower on the third day of treatment. However, on the fourth day, scores of erythema and scaling of both groups are approaching to the same level. Cumulative scores showed the same trend. There was no difference in the thickness scores between the two groups. Then we further measured the thickness of epidermis, using H&E stained skin sections from WT and *Nrip1−/−* mice, both in IMQ treated and untreated areas. The H&E stained sections were digitally imaged, then the thickness of epidermis was measured in 10 microscopic fields (40x) for each core in blind studies conducted by two researchers, one of which is a clinical dermatologist. As the results showed in Figure 4C, after 5 days of IMQ treatment, thickness of epidermis in treated area was increased in both two groups, but the increment was significantly reduced in *Nrip1^−/−^* mice, compared to WT mice.

### The depletion of Nrip1 suppresses the IMQ-induced expression of p65

**Figure 5:**
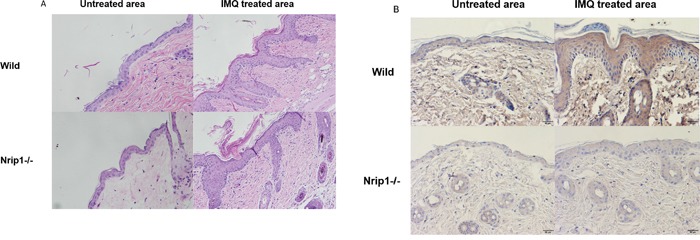
Histology and immunohistochemical (IHC) staining of IMQ treated lesion **A.** H&E (200×), two genotypes of mice showed typical features of psoriasis, including hyper-proliferative keratinocytes, parakeratosis and the absence of granular layer, but the density of epidermal ridge and inflammatory cell infiltration in the dermis of *Nrip1^−/−^* mice were much less than WT mice. **B.** IHC (200×), p65 positive cells within IMQ treated skin were decreased in both epidermis and dermis of *Nrip1^−/−^* mice.

Analysis of H&E-stained sections of IMQ-treated skin from these two different genotypes of mice showed typical characteristics of psoriasis biopsy (Figure 5A), such as hyper-proliferative keratinocytes, parakeratosis and the absence of a granular layer. However, the inflammatory cell infiltration in the dermis of Nrip1−/− mice was much less than that in WT mice, which suggested that the depletion of Nrip1 may reduce inflammation caused by IMQ.

In order to detect the function of Nrip1 deficiency on NF-κB during IMQ treatment, IHC staining was used to detect the expression of p65 in skin sections from WT and *Nrip1−/−* mice, both in IMQ treated and untreated area. The p65 stained sections were digitally imaged. The IHC results (Figure 5B) showed that compared to WT mice, p65 positive cells within IMQ treated skin were decreased in epidermis and dermis of *Nrip1^−/−^* mice, respectively.

## DISCUSSION

NRIP1 interacts as a co-activator or co-repressor with numerous nuclear receptors, including peroxisome proliferatoractivated receptors (PPARs), liver X receptor (LXR), estrogen receptor-related receptor (ERR), and estrogen receptor (ER) [2]. A recent study revealed that NRIP1 may stimulate the activity of NF-κB by forming a trimeric complex with RelA and CBP [4], and upregulate downstream inflammatory genes, including TNF-α and IL-6, both of which play crucial roles in the pathogenesis of psoriasis. Other studies have shown that except for macrophages, NRIP1 may also work as a coactivator of pro-inflammatory gene expression in adipocytes and possibly other cell types [11].

In the current study, we investigated the role of NRIP1 in the pathogenesis of psoriasis, including cell proliferation, apoptosis and inflammation. Our research revealed for the first time that *NRIP1* mRNA was elevated in the skin lesion, which is in accordance with our previous data of immunohistochemistry showing that expression of NRIP1 was significantly increased both in the epidermis and dermis of PP (data unpublished). Abnormal proliferation of keratinocytes is a important mechanism of psoriasis, resulting in epidermal hyperplasia, a morphological characteristic of psoriasis [12]. Numerous well-established anti-psoriatic treatments, including phototherapy, oral retinoid and topical calcipotriol, could improve the condition of psoriasis patients via influencing keratinocytes proliferation [13]. So, we performed an *in vitro* study with HaCaT cells to detect the role of NRIP1 in keratinocytes proliferation. Our data showed that suppression of NRIP1 in HaCat cells could significantly inhibit cell growth and induce apoptosis, suggesting that the suppression of cell growth may due to the induction of apoptosis, and indicating that NRIP1 may play a role in cellular proliferation and apoptosis of keratinocytes involved in psoriasis. Our previous study also showed that in breast cancer cells, suppressing NRIP1 could reduce cells proliferation and induce apoptosis [7], however, another study found that overexpression of NRIP1 could reduce the proliferation of colon cancer cells [14]. These contradictory results of NRIP1 indicate that NRIP1 may play diverse roles on cell proliferation in different cell types. So, we need to further confirm our findings on human primary keratinocytes.

With advances of psoriasis pathogenesis, psoriasis is considered as an autoimmune disease, which is centrally controlled by T cells [15], interplaying with numerous cell types via different cytokines, such as NF-κB [8]. NF-κB is a key element regulating inflammation pathways, and it links altered immune cell states and keratinocytes in the pathogenesis of psoriasis [16]. Our data firstly showed that *NRIP1* mRNA was increased in PBMCs of psoriasis patients compared to healthy controls. Then we confirmed that the expression of *RelA/p65* was elevated in lesions and PBMCs of psoriasis patients, which was in accordance with other previous studies [9]. In order to evaluate the effect of NRIP1 on NF-κB, we detected the expression of p65 in HaCaT cells and primary CD4^+^ T cells. The results revealed that suppressing NRIP1 in HaCaT cells and CD4^+^ T cells could significantly downregulate the expression of RelA/p65. These data suggested that NRIP1 might upregulate the secretion of downstream cytokines by stimulating the expression of *RelA/p65* in CD4^+^ T cells in psoriasis. IL23/Th17 axis was strongly supported to play predominant roles in the pathogenesis of psoriasis in recent studies, studies have showed that the IL-23 pathway is activated and the expression of IL-23 and IL-17 were higher in psoriasis patients than healthy controls [17]. The IL-17 level was correlated with the severity of psoriasis [18], and therapies targeting IL-23 and IL-17 had been evaluated with promising effect [19]. In this study, suppression of NRIP1 in CD4^+^ T cells also decreased the secretion of IL-17 significantly, suggesting that NRIP1 may involve in psoriasis via upregulating IL-17.

The *in vivo* data suggested that IMQ-induced skin inflammation was delayed in *Nrip1^−/−^* mice compared to WT mice during the first three days of treatment. The scores of erythema and scaling, and thickness of epidermis of *Nrip1^−/−^* mice were all decreased before the 3rd day. And histological analysis of IMQ-treated skin showed that the density of epidermal ridge and inflammatory cell infiltration in the dermis of *Nrip1^−/−^* mice were much less than in WT mice, which suggested that the depletion of Nrip1 may reduce inflammation caused by IMQ treatment. However, on the last day of IMQ treatment, both types of mice had developed similar lesions resembling psoriasis. Thus, we need to further investigate the lesion features on the 3rd day of IMQ treatment, when the lesions of Nrip1^−/−^ mice seem to be obviously weaker than those on the WT mice. The IHC results revealed that the expression of p65 in IMQ-treated skin was remarkably reduced in the epidermis and dermis of *Nrip1^−/−^* mice compared to WT mice, which was in accordance with the data that NRIP1 suppression could downregulate expression of p65 in patients' CD4^+^ T cells. These results were attractive, because p65, one important subunit of NF-κB, could suggest the involvement of NF-κB to a certain extent, although we need to further detect the active status of NF-κB in order to confirm the role of NRIP1 on NF-κB.

As IMQ is a ligand of TLR7 and TLR8 [20], it is also an intense irritant which can induce an obvious inflammatory reaction on skin [21]. The depletion of NRIP1 may only cause a partial decay of pro-inflammatory gene transcription [3]. In other words, NRIP1 may play a role in a subtle state of inflammation, which means NRIP1 deficiency alone is not sufficient to impair the intense inflammatory response to IMQ. This may be the reason that the *Nrip1^−/−^* mice developed weaker lesions only during the first three days of IMQ treatment. However, the RelA/p65 expression in the epidermis and dermis of the IMQ treated area was remarkably reduced in *Nrip1^−/−^* mice, suggesting that the depletion of NRIP1 could inhibit the expression of RelA/p65, and also verifying the previous results of human skin, PBMCs, primary keratinocytes and CD4^+^ T cells.

In conclusion, our studies reveal that NRIP1 might play roles in cellular proliferation and apoptosis of keratinocytes, activation of CD4^+^ T cells and NF-κB gene transcription, which are all involved in the pathogenesis of psoriasis, and suggest that NRIP1 may be a novel and attractive therapeutic target for psoriasis. However, more detailed studies are needed to confirm the mechanism behind the function of NRIP1 on inflammation and cell growth and to explore its other potential roles in psoriasis.

## MATERIALS AND METHODS

### Skin and blood samples

Thirty psoriasis patients in progressive status with plaque lesions and 16 healthy volunteers were enrolled for this study. Their characteristics are summarized in Table 1. Two 5 mm punch biopsies of PP and PN from each patient, as well as one 5 mm punch of NN, were obtained. Five ml peripheral blood was also obtained from each donor. The diagnosis of psoriatic patients was performed by a consultant dermatologist, who also provided patient demographic information and disease parameters along with psoriasis area and severity index (PASI). Healthy controls were defined as people who had no history of psoriasis and no skin lesions on clinical examination. Each member of the healthy control group had a qualified match in age and sex with the psoriatic patient group. All samples were collected in accordance with the ethical guidelines mandated by the ethical committee of the Institute of Dermatology, Chinese Academy of Medical Sciences. Informed consent was signed by those who agreed to participate in this study. All skin biopsy specimens were immediately stored in liquid nitrogen for real time quantitative polymerase chain reaction (qRT-PCR) experiments.

**Table 1:** Demographics and disease parameters of patients with psoriasis

Case	Sex	Age (years)	Nail involved	Family history	Lengh of disease (years)	PASI score
1	F	45			3	46.2
2	M	33		+	5	35.4
3	F	50			10	5.8
4	M	41	+	+	10	16.2
5	F	22			1	8.4
6	M	33			3	36.3
7	M	35	+		11	9.4
8	M	34	+		6	11.6
9	M	26			1	20.2
10	M	52			2	26.3
11	M	32		+	1	15.2
12	M	23			8	14.8
13	M	44			7	5.2
14	M	50	+	+	10	9.2
15	M	20			8	5.4
16	M	51	+	+	6	16.3
17	M	59			10	26.4
18	M	48			5	15.5
19	M	48			8	22.6
20	M	51			8	25.5
21	M	25			1	12.4
22	M	48			20	8.5
23	M	25	+		1	15.5
24	M	25	+		1	26.3
25	F	48			3	8.6
26	F	28			1	14.4
27	F	25		+	10	4.3
28	F	22			2	16.2
29	F	51		+	19	20.5
30	F	47			3	8.6

### HaCaT cells

HaCaT cells were cultured in Dulbecco's modified Eagle's medium (DMEM) supplemented with 10% fetal bovine serum (FBS), 100 U/ml penicillin, and 100 μg/ml streptomycin, and incubated at 37 °C in a humidified atmosphere containing 5% CO2.

### NRIP1 shRNA transfection

HaCaT cells were seeded in a six-well plate with 60% of confluence in DMEM with 10% FBS and polybrene (8 μg/ml). Lentiviral particles (20 μL/mL) containing shRNA of human NRIP1 (Santa Cruz, CA) were added to the cells and incubated for 24 h, then medium was replaced with fresh growth medium, cells were cultured for another 24 h. Puromycin (0.25 μg/mL; Sigma-Aldrich) was used to select the stable clones expressing target shRNA. The culture medium was changed every 2–3 days, until resistant colonies could be identified. Non sense shRNA (shCON) lentiviral particles (Santa Cruz, CA) were added to the control cells. The expression of NRIP1 in stable cells was detected by qRT-PCR.

### MTT assay

To evaluate the viability of shRNA treated HaCaT cells, at 24, 48, 72, 96 and 120h after being seeded in 6-well plates (5×10^5^ cells/well), 100 μl of 5mg/ml 3-(4,5-dimethylthiazol-2-yl)-2,5-diphenyltetrazolium bromide (MTT, Sigma, St. Louis, MO) and 1.9 ml serum-free culture medium were added to each well. Following a four-hour incubation, wells were aspirated and 2 mL of DMSO was added to each MTT-treated well. Each well was divided into 10 wells of a 96-well-plate, and the absorption at 540 nm was measured by spectrophotometry (BioTek PowerWave XS, Winooski, VT, US). An MTT assay for each cell line was repeated 3 times.

### Propidium iodide (PI) staining

For cell cycle and apoptosis analysis, HaCaT cells were transfected with shRNA as described above, then were trypsinized and resuspended in PBS with 0.1% bovine serum albumin. A total of 1×10^6^ cells were fixed in 25% ethanol overnight at 4°C. The cells were then stained with PI (50 mg/mL) containing RNase A (0.7 mg/mL), and incubated at 37°C. 10,000 events were collected for each sample, and examined by flow cytometry (BD Accuri C6, Franklin Lakes, NJ). Each group was assayed three times.

### Annexin-V staining

shRNA induced apoptosis in HaCaT cells was verified and evaluated by Annexin-V staining. The cells were stained with PE Annexin-V and 7-Amino-Actinomycin (7-AAD) following the manufacturer's instructions (BD Pharmingen, Franklin Lakes, NJ) to detect early apoptosis cells (PE Annexin- V^+^/7-AAD^−^events) and late apoptosis cells (PE Annexin- V^+^/7-AAD^+^events) and examined by flow cytometry (BD Accuri C6). Apoptosis of each group was assayed three times.

### Western blot analysis

About 1 × 10^6^ HaCaT cells were lysed in RIPA Lysis Buffer for 30 min, then collected and measured by using the BCA Protein Assay Reagent Kit (Pierce, Biotechnology). Equal amounts of protein from each sample were mixed with 1× loading buffer and denatured at 95 °C for 5 min, then they were subjected to 10% SDS-PAGE and transferred on to polyvinylidene difluoride membranes (Roche, Germany). After being blocked for 2h with 5% bovine serum albumin in 0.1% Tween-20/TBS, the PVDF membranes were incubated with primary antibody against Human p65 (Abcam, UK) at a concentration of 1:1000 at 4°C overnight. After being washed, the membranes were incubated with HRP-conjugated secondary antibody at 1: 3000 dilutions at room temperature for 2h. After being washed again, protein bands were detected by enhanced chemiluminescence. The images were acquired by FluorChem FC2 System (Alpha Innotech Corporation, USA). The membrane was stripped and reprobed with GAPDH antibody.

### CD4^+^T cells

Firstly, PBMCs were isolated from peripheral blood of psoriasis patients by Ficoll-Paque density gradient centrifugation, and then CD4^+^T cells were isolated using BD IMag ™ human CD4^+^T cells enrichment set according to manufacturer's protocol (BD, USA). Briefly, for every 10^7^ cells, 50 μl BD IMag ™ CD4^+^ beads were added, mixed thoroughly, and incubated at room temperature for 30 mins. Following that, 1ml BD IMag ™ buffer was added, and cells were transferred into FACS tubes and placed in strong magnetic field for 8-10 min. CD4^+^T cells were then attached onto the internal wall of the tubes. The supernatants were carefully removed, and then the tubes were placed out of the magnetic field and washed twice by using the IMag ™ buffer. Finally, the supernatants were discarded and the remaining cells were collected for the following experiments.

### NRIP1 siRNA transfection

The RNA interference (siRNA) sequence for silencing human NRIP1 was purchased from Genepharma (Shanghai, China). The sequences are as follows: siNRIP1: FP-5′GAGGAUCAGAACUUUAACATT3′, RP-5′UGUUAAAGUUCUGACCUCTT3′. Negative control of siRNA (siCON) was transfected as matched control. Transfection of the siRNAs were performed using Lipofectamine® 2000 (Thermo Fisher Scientific, Waltham, MA, USA) according to the manufacturer's protocol. CD4^+^ T cells were seeded at a density of 3×10^5^ cells/well in 6-well plates, when the cells reached 60-80% confluency, the siRNAs- Lipofectamine® 2000 complexes were prepared and added to each well. After 6h of incubation, the medium was replaced with fresh culture medium. The medium was replaced with 2 ml fresh pre-warmed medium per well every 24 h. After 48 h of siRNA transfection, the cells were harvested for qRT-PCR.

### qRT-PCR

Total RNA was extracted from 2×10^6^ cells using TRIZOL (Invitrogen/Thermo Fisher Scientific, Waltham, MA, USA) according to the manufacturer's protocol, and the quantity of RNA was determined by a spectrophotometer at 260 nm. 20 μl of cDNA was synthesized from 1 μg of the total RNA using RevertAid™ First Strand cDNA Synthesis Kit (MBI Fermentas, Ontario, Canada). qRT-PCR analysis was performed with SYBR® Premix Ex Taq™ (TaKaRa Co., Dalian, China). Following the manufacturer's protocol, 1 μL RT cDNA was mixed with 0.6 μL forward, 0.6 μL reverse primers (10 μM), 10 μL Premix Ex Taq™, and 8.4 μL Nuclease-free water to obtain a final volume of 20 μL. qRT-PCR reactions were run on an Applied Biosystems 7300 Real-Time PCR System (Applied Biosystems, Foster City, CA, USA). The amplification conditions were 95 °C for 10 min, and 40 cycles of 95 °C for 30 s, 60°C for 30 s and 72 °C for 30 s. The levels for target gene mRNA were normalized to GAPDH in the same sample. For NRIP1 primer, forward, 5′- GCTGGGCATAATGAAGAGGA-3′, reverse, 5′- CAAAGAGGCCAGTAATGTGCTATC-3′. For *RelA/p65* primer, forward, 5′-CTGCAGTTTGA TGATGAAGA-3′, reverse, 5′-TAGGCGAGTTATAGCC TCAG-3′. For *GAPDH* primer, forward, 5′- ATGGG GAAGGTGAAGGTCG-3′, and reverse, 5′- GGGGT CATTGA TGGCAACAATA-3′. qRT-PCR assays for each sample were repeated 3 times.

### Enzyme-linked immuno sorbent assay (ELISA)

CD4^+^T cells were transfected with RNA interference (siRNA) sequences and supernatants were collected at 48h and 72h after transfection. The concentration of IL-17 was measured by using ELISA kits (ELH-IL1, 7RayBiotech, GA, USA). One-hundred μl of each standard and supernatant sample were added into the 96-well plate coated with anti-Human IL17 and incubated over night at 4°C with gentle shaking, and washed 4 times with washing buffer. Wells were incubated for 1 hour with IL-17 specific biotinylated antibody, rinsed 4 times with wash buffer, reacted with streptavidin solution diluent for 45 minutes, washed 4 times with wash buffer, and then incubated for 30 minutes with 100 μl TMB One-Step substrate reagent in the dark. The plates were quenched with stop solution and the absorption at 450 nm was measured by spectrophotometry (BioTek PowerWave XS, Winooski, VT, US). ELISA assays for each sample were repeated 3 times.

### Mice and treatments

10- to 12-month-old C57BL/6J (B6) and *Nrip1^−/−^* mice were used. The Nrip1 knockout mice had been backcrossed to B6 for more than 10 generations before the homozygous knockout (*Nrip1^−/−^*)** mice were generated. The animal housing conditions are described previously [7]. Animal care and handling were conducted according to NIH guidelines and the policies of Southern Illinois University School of Medicine Laboratory Animal Care and Use Committee, Springfield, IL. Both groups of mice received a daily topical dose of 62.5 mg IMQ cream (5%) (Aldara; 3M Pharmaceuticals) on their shaved backs for 5 consecutive days.

### Scoring severity of skin inflammation

We scored the severity of inflammation of the back skin based on the clinical Psoriasis Area and Severity Index (PASI) [20]. And we scored erythema, scaling, and thickening independently on a scale from 0 to 4, and the cumulative score from 0 to 12.

### Histology and IHC assay

Samples from back skin (3 mm diameter) were excised and fixed overnight in 10% neutral buffered formalin, then transferred to PBS (pH 7.4), and embedded in paraffin. Sections (4 μm thick) of each specimen were cut for H&E staining and IHC study.

Tissue slides were deparaffinized with xylene and then rehydrated with decreasing ethanol concentrations. Antigen retrieval was conducted by boiling-bath method in 0.01 M sodium citrate buffer, pH 6.0 to about 95°C, and then slides were put in the buffer for 15 min. Blocking solution was used to prevent nonspecific binding of antibodies. Sections were incubated with polyclonal anti-NRIP1 antibody (SCBT, Santa Cruz, CA, 1:50 dilution) at 4°C overnight. HRP Detection System (Dako EnVision System HRP; Dako North America, Inc. Carpinteria, CA), was used for detection. After counterstaining with hematoxylin (Harris Modified Hematoxylin, Fisher Scientific, Fairlawn, New Jersey), the sections were dehydrated and mounted. The specific staining of NRIP1 in the sections was examined microscopically (Olympus, Center Valley, PA, USA).

### Statistical analysis

All data were analyzed with Prism 5.0 (GraphPad Software Inc., San Diego, CA, USA) and expressed as means ± SE. T-test and one-way analysis of variance (ANOVA) were used for data analysis. Differences with P < 0.05 were considered significant.
